# The weight of caring for your elderly – a cross-sectional analysis of non-professional caregivers for people living with dementia in Romania.

**DOI:** 10.1192/j.eurpsy.2023.1854

**Published:** 2023-07-19

**Authors:** S. Zaharia, T. Alexandrescu, T.-C. Ionescu, C. Tudose

**Affiliations:** 1 “Prof. Dr. Alexandru Obregia” Clinical Psychiatric Hospital; 2 “Carol Davila” University of Medicine and Pharmacy, Bucharest, Romania

## Abstract

**Introduction:**

While a growing corpus of literature regarding the stress suffered by caretakers for people living with dementia (PLWD) already exists, very little data is available regarding this subject among Romanian caretakers.

**Objectives:**

This cross-sectional study aims to compensate for this by assessing a small (N=72) sample of caretakers through the use of self-reporting questionnaires for subjective feelings of stress and burden.

**Methods:**

Responders filled and online survey containing miscellaneous socio-demographic questions and the Kingston Caregiver Stress Scale (KCSS) along with the Caregiver Health Assessment Self Questionnaire (CHASQ). Results were collected and analysed in SPSS for subsequent correlations.

**Results:**

The majority (77%) of caretakers are women and 86% of responders are offering their care at home, emphasizing pervasive gender roles and lack of availability or accessibility of social services for the PLWD in Romanian society. Three thirds of caregivers were children of PLDW. More than half of responders (51%) had KCSS scores that suggested severe stress while less than 9% related only mild stress. Most responders (52%) related social aspects of their lives as most affected by their caregiver status.

**Conclusions:**

While in line with most other findings and limited in scope and means by its methodology, this study offers a quick snapshot on the subjective levels of stress affecting caretakers of Romanian PLWD and can lead towards further points of inquiry on the matter in the Romanian population.

**Disclosure of Interest:**

None Declared

